# A Bridging: Distraction External Fixation for a Severely Comminuted Fracture of the Middle Phalanx. A Case Report

**DOI:** 10.7759/cureus.4007

**Published:** 2019-02-04

**Authors:** Ioannis Galanopoulos, Georgios Vynichakis

**Affiliations:** 1 Orthopaedics, Thriasio General Hospital, Athens, GRC; 2 Orthopaedics, General Hospital of Piraeus Tzaneio, Piraeus, GRC

**Keywords:** middle phalanx fractures, comminuted, bridging external fixation, ligamentotaxis

## Abstract

Severely comminuted fractures of the middle phalanx of the digits are known to be demanding cases for hand surgeons. The restoration of the length and axis of the phalanx, such as the anatomic reduction of the articular surface in case of intra-articular association are basic principles that have to be followed. A simple and cheap device consisting of two Kirschner wires and a nylon suture can work as an external fixation system based on the principle of ligamentotaxis. As a result, the anatomic reduction of the fracture with minimal soft tissue damage and simple surgical equipment is feasible.

## Introduction

Complex hand injuries become increasingly common because of work, motor vehicle accidents, and sports activities. They may include not only fractures but also open wounds with various degree of soft tissue damage. The combination of a comminuted, open fracture of the middle phalanx with severe soft tissue damage limits the treatment options and any procedure should be carefully planned [1]. An external fixation system based on the principle of ligamentotaxis can give a good outcome, with minimal additional tissue trauma [2]. The surgeon can achieve anatomical reduction quickly and with minor effort. Additionally, only minimum surgical equipment and a digital nerve block are necessary for the operation [3].

## Case presentation

**Figure 1:**
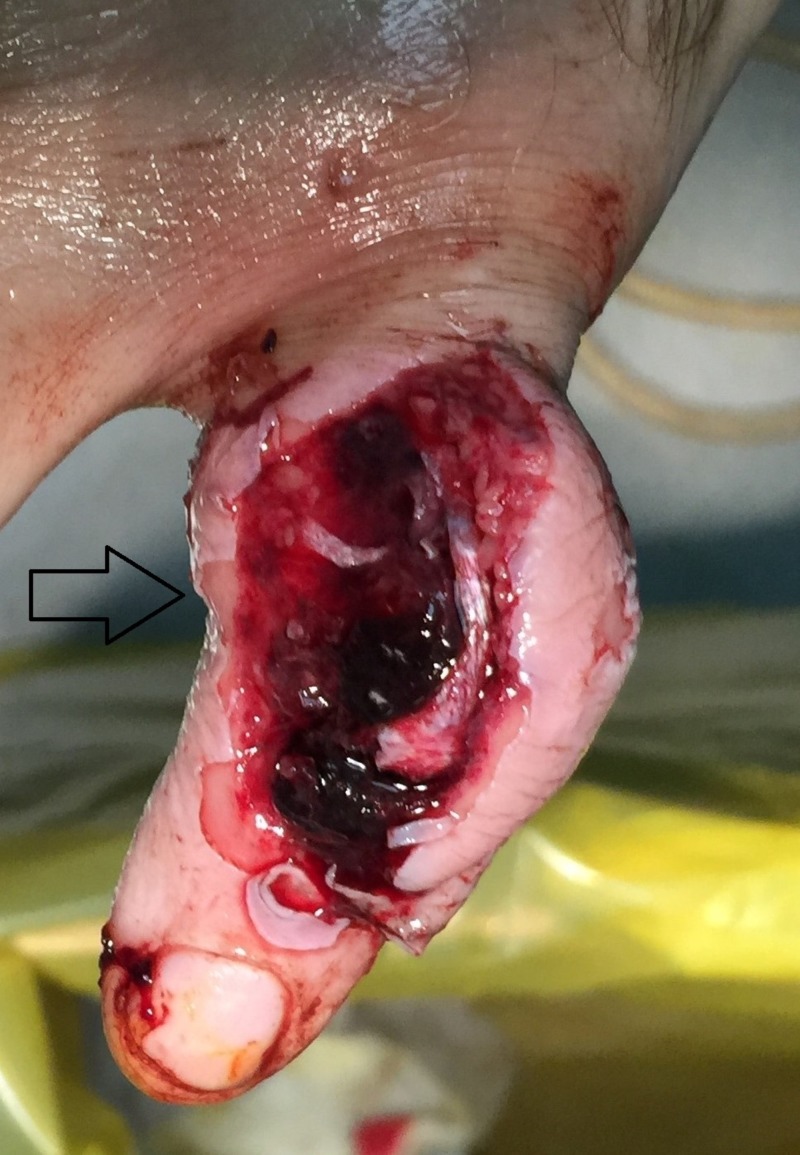
Crush injury of the middle phalanx with an open wound

**Figure 2:**
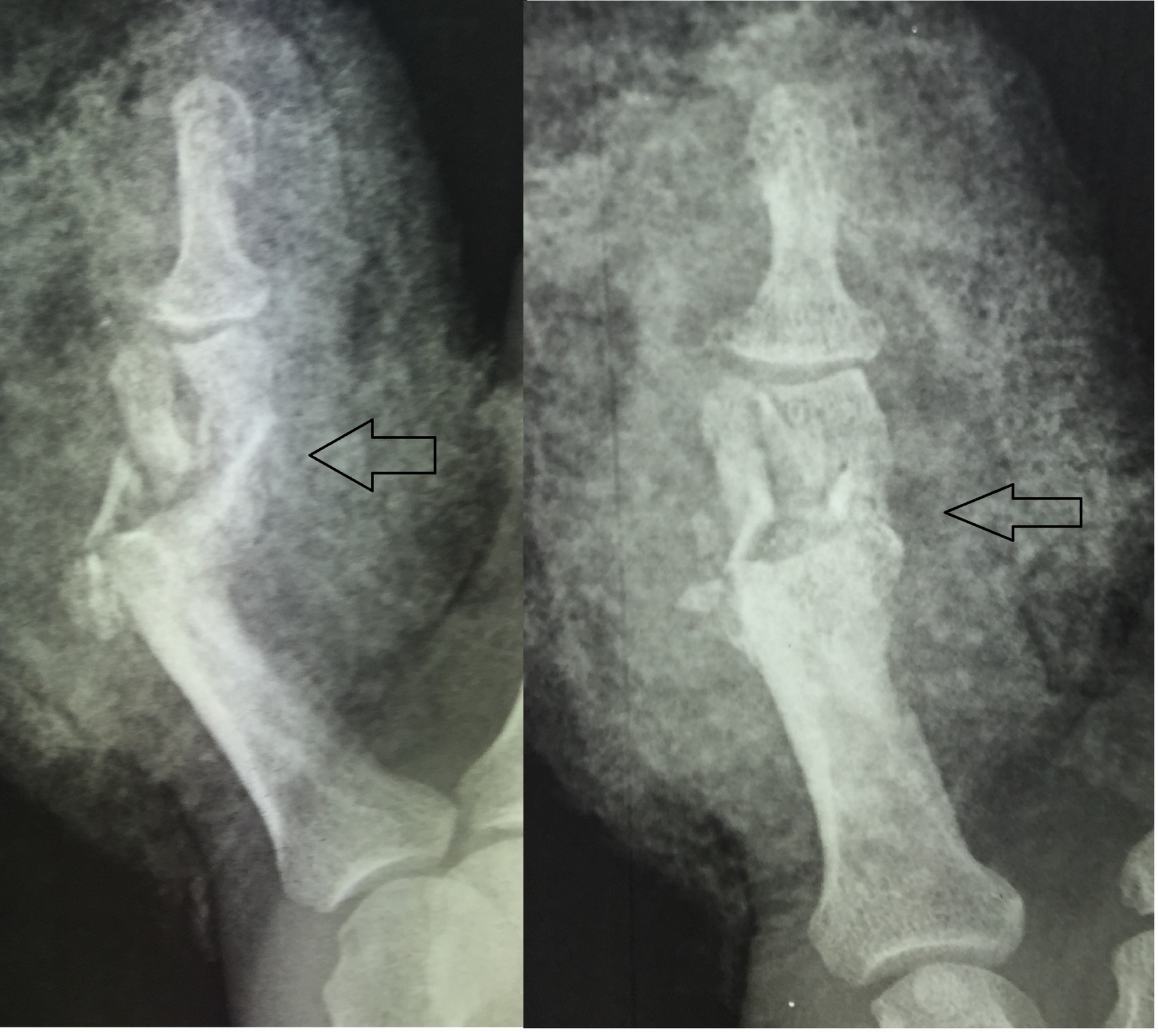
Plain radiographs: severely comminuted, intra-articular fracture of the middle phalanx

**Figure 3:**
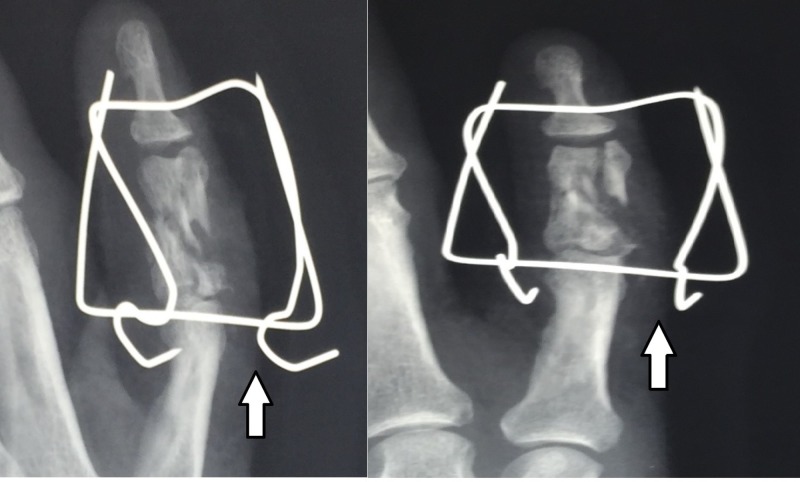
Reduction of the fracture due to the ligamentotaxis principle

**Figure 4:**
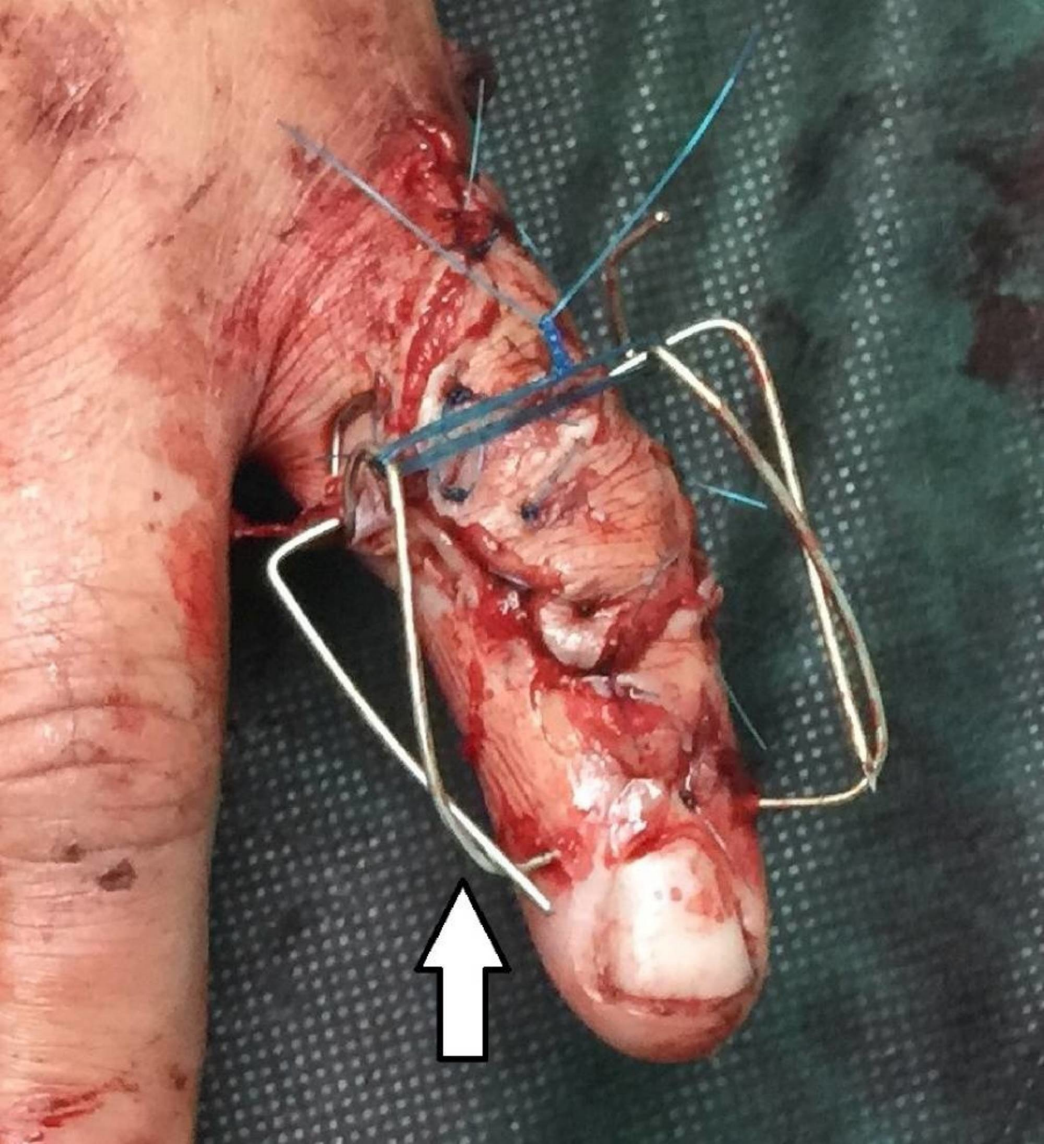
Final clinical picture

The patient is a 50-year-old, right-handed, male laborer who suffered a crush injury of the left hand (Figure 1). He suffered from an open, severely comminuted fracture of the middle phalanx of the small finger with extensor mechanism disruption. The fracture extended to both the proximal and distal articular surfaces (Figure 2). The neurovascular function of the digit was normal. Immediately, a dose of first-generation cephalosporin and clindamycin was given. On a standard hand table, with the patient in the supine position, with regular draping and detailed irrigation, a digital nerve block was performed in order to proceed to the operation. After careful debridement, two K-wires, as previously described, were inserted, bent, and secured. The fracture was reduced due to the principle of ligamentotaxis (Figure 3). After that, the extensor mechanism of the digit was repaired with non-absorbable sutures and the skin was closed appropriately (Figure 4). The patient remained hospitalized for 48 hours for intravenous antibiotics and he continued with oral antibiotics until seven days postoperatively. He had a follow-up at one, two, and four weeks postoperatively and, after that, at eight weeks when the fracture was fully healed and the device removed. He started a rehabilitation program with slight mobilization and for six weeks after the device removal, work and sports activities were not permitted to avoid a new hand injury. Six weeks after the device removal, he fully returned to work and daily activities.

## Discussion

As in every fracture case, the surgeon has to follow the general principles of reduction and fixation. Many different techniques have been described in the literature for middle phalanx fractures. For simple, transverse fractures, the open reduction and internal fixation with a plate, as well as intramedullary fixation with k-wires or cannulated screws, can be reliable treatment options [4-5]. For more complex, comminuted fractures, external fixation methods that achieve reduction based on the principle of ligamentotaxis seem to be more efficient [6-8].

Intra-articular fractures are challenging cases for hand surgeons and anatomic reduction is necessary for a good outcome with respect to movement and function. In these cases, external fixation systems may give a good outcome with minimal tissue trauma [9].

Severely comminuted fractures of the middle phalanx, with or without the involvement of the articular surface and various types of soft tissue damage, are indicated for external fixation with continuous distraction [10].

After skin preparation and sterile surgical draping, the surgeon proceeds to a digital nerve block with lidocaine 1%. In case of soft tissue trauma or an open wound, detailed irrigation and debridement follow. Two parallel k-wires are inserted in the distal part of the proximal phalanx and the proximal part of the distal phalanx, respectively. The k-wires must be parallel to the rotational axis of the interphalangeal joints. After that, the surgeon bends the distal k-wire, making an arrow. This arrow works as a spring and gives force to the distal k-wire all the time. This technique allows dynamic fixation. After the ligamentotaxis and fracture reduction, a non-absorbable suture (nylon 2-0) is inserted to connect the two k-wires. The suture secures the connection between the k-wires. A soft bandage is placed around the digit. This construct is based on the "Suzuki frame" technique and constitutes a modification of it [11].

The surgeon gives detailed instructions to the patient to keep the construct clean, particularly on the skin insertion points. The patient has a follow-up with plain radiographs in two weeks postoperatively to check the fracture reduction. After that, new plain radiographs in six and eight weeks postoperatively are necessary to evaluate the healing process. After the full healing of the fracture, the k-wires are removed and a protocol of sight mobilization begins. Any physical activity that could repeat the injury of the finger is avoided for six weeks after the removal of the k-wires.

Potential pitfalls include inadequate k-wire bending and suture untying. Inadequate bending of the k-wire does not allow the structure to work with continuous traction and, as a result, the principle of ligamentotaxis cannot be utilized. Additionally, the suture untying makes the construct unsecured and it may be destructed. Complications like a pin-tract infection may also be possible due to poor device cleaning.

## Conclusions

This simple construct made with two k-wires and a nylon suture has very good outcomes and very low cost in case of severely comminuted fractures of the middle phalanx using the principle of ligamentotaxis.
